# Modeling Analysis of Complex Deformation of Woven Coating Film during the Cyclic Tensile Process

**DOI:** 10.3390/polym16121623

**Published:** 2024-06-07

**Authors:** Li Cai, Zhengyan Zhang, Deng’an Cai, Guangming Zhou, Xinwei Wang

**Affiliations:** 1 State Key Laboratory of Mechanics and Control for Aerospace Structures, Nanjing University of Aeronautics and Astronautics, Nanjing 210016, China; 2 Engineering Faculty, University of New South Wales, Sydney NSW 2052, Australia

**Keywords:** woven coated film, cyclic stretching, constitutive model, intelligent inverse algorithm, single-objective genetic algorithm, numerical simulation

## Abstract

It is difficult for the existing Burgers model to accurately depict the off-axis cyclic drawing process of woven coatings. In this paper, the mechanical deformation of woven PVC (polyvinyl chloride)-coated film at different temperatures is investigated. One-dimensional (1D) and two-dimensional (2D) constitutive models were established to characterize cyclic deformation processes. The 1D model is an improved Burgers model. The effects of the time dependence of the viscosity coefficient and the ratio of elastic to viscous deformation are considered simultaneously. The accuracy of the 1D model for predicting the cyclic nonlinear deformation at different temperatures and loading rates is improved. The 2D model is a nonlinear orthotropic model using polynomials. On the basis of the single-objective genetic algorithm, the inverse algorithm is used to obtain the shear polynomial coefficients in the tension phase and the shear modulus in the unloading phase, which circumvents performing the difficult shear test. UMAT subroutines of off-axis stretching and off-axis cyclic stretching are written separately. The intelligent inverse algorithm program consists of a single-objective genetic algorithm program, a finite element parametric modelling program, and a UMAT subroutine. The simulation results are compared with the off-axis cyclic tensile test data to validate the effectiveness and accuracy of the proposed 2D model for the analysis of the woven PVC-coated films in the tension–shear coupling state.

## 1. Introduction

Woven coating film material has been widely used in aerospace, construction, and other industries because of its excellent flexibility, easy cutting, fast installation, large covering space, and other engineering advantages [1,2,3,4,5,6]. At present, researchers have made many achievements in the theoretical research, structural analysis, and form-finding research of coated film materials [7,8,9,10]. The mechanical properties of the coated film seriously affect the application of the membrane structure in engineering, and it is necessary to investigate the mechanical characteristics of the coated film systematically.

Different from resin matrix composites, the mechanical analysis of the membrane is a highly nonlinear problem. Therefore, accurate modeling is the primary premise of mechanical behavior analysis of membrane structure. Xu et al. [11] used an improved viscoelastic model to analyze the influence of prestressing on the impact properties of PVDF (polyvinylenedifluoride)-coated fabrics. Xu et al. [12] studied the influence of damping characteristics on the dynamic response of the coated film. To study the vibration characteristics of coated films, Li et al. [13] established a random vibration model based on von Karman’s large deflection theory. The effects of impact velocity and preload on the reliability of membrane structures are studied by using the new vibration model. For the study of tear performance of coated film, Zhang et al. [14] established a multiphase tear expansion model based on experimental data. Compared with other models, the new model was more accurate in predicting the tear strength. Based on the theory of fracture mechanics, Sun et al. [15] proposed a new tear residual strength model to analyze the tear deformation of woven coating film during the tensile process. He et al. [16] developed a new parameter acquisition method, the virtual crack closing technique (VCCT), to obtain the fracture parameters of woven PTFE (polytetrafluoroethylene)-coated film. The new method improves the prediction accuracy of the model for crack propagation.

When the coating film is exposed to the external environment for a long time, its material structure and mechanical properties will change. Li et al. [17] used an improved fiber drawing toughness model to analyze the influence of different environmental factors on the aging properties of coated films. On the models of the cyclic deformation of coated fabrics, Zhang et al. [18] adopted a new viscoelastic model to predict the cyclic deformation of PTFE-coated fabrics. However, their new model was not accurate in predicting the nonlinear deformation during unloading, mainly because the new model did not take into account the nonlinear effect of the coefficient of viscosity. Meanwhile, Xu et al. [19] proposed a segmented phenomenological model to analyze the cyclic mechanical properties of coated fabrics. Dib et al. [20] developed a new time-independent and irreversible model, taking both the anisotropy and viscoplasticity of coated fabrics into consideration. The new constitutive model was implemented in ABAQUS 2021 to describe the tensile behavior of coated fabric under cyclic load. In contrast, Xu et al. [21] presented a time-dependent model to analyze the biaxial creep deformation of PVDF-coated fabrics. Based on the experimental data of uniaxial and biaxial stretching, Dinh et al. [22] established a segmented elastic-plastic model to predict the deformation of uniaxial cyclic tensile and biaxial cyclic tensile of PVC-coated film. The new model can capture the basic features of cyclic stretching, but it cannot depict the nonlinear behavior of the coated film during cycling. Moreover, the model parameters need to be obtained from uniaxial and biaxial tensile tests.

Genetic algorithms are increasingly used in composite structures due to their powerful analytical capabilities. To solve the composite delamination phenomenon of airship propellers, Meng et al. [23] performed both local optimization and global optimization to enhance the stiffness of the propeller through genetic algorithm. Based on the multi-objective genetic algorithm, Yang et al. [24] designed and optimized the structure of a biaxial tensile test piece of carbon fiber laminates so that the failure occurred at the middle position. Bai et al. [25] obtained the best practice of a genetic algorithm by using the quantitative analysis method, and they optimized the stiffness and weight of woven composite springs. Pourrajabian et al. [26] used the continuous genetic algorithm and binary genetic algorithm to optimize wind blades, and they compared the accuracy of both. It was found that the accuracy and efficiency of the continuous genetic algorithm was higher than that of the binary genetic algorithm. Moreover, the optimization rate of the binary genetic algorithm can be improved using uniform crossover. It can be seen that the existing studies are mainly about the structural optimization of composite materials by the genetic algorithm. There are few studies focused on obtaining the material parameters of models using genetic algorithms.

Many modeling analyses of coating film exist, but the one-dimensional (1D) modeling research on cyclic drawing is rare. For the cyclic tensile deformation of the coated film, the existing Burgers model does not simultaneously consider the effects of the time-dependence of the viscosity coefficient and the ratio of elastic to viscous deformation during cycling. Moreover, the prediction accuracy of the existing orthotropic models is low for the cyclic tensile behavior of the coated film in the tension–shear coupling state. In the cyclic tensile process, all material parameters of the orthotropic model are obtained from the test data, and intelligent genetic algorithms are rarely used to obtain model parameters. Therefore, further research is needed to address these issues.

In this paper, a 1D model and 2D model are proposed to investigate the uniaxial cyclic tensile deformation of woven PVC-coated films at different temperatures. The 1D model, called the improved Burgers model, is a viscoelastic model and is developed based on the Burgers model. The optimization coefficients are introduced in the improved Burgers model, and the time-dependent effect of the viscosity coefficient is also considered. The optimization coefficient adjusts the proportion of elastic and plastic deformation in the tensile process, and the viscosity coefficient considering the time-dependence effect enhances the analytical accuracy of the improved Burgers model for nonlinear deformation and thus improves the prediction accuracy of the Burgers model for nonlinear deformation at different temperatures and loading rates. The 2D model is a nonlinear orthotropic model using three polynomials. On the basis of a single-objective genetic algorithm, the inverse algorithm is used to obtain the shear polynomial coefficients in the tension phase and the shear modulus in the unloading phase, which circumvents performing the difficult shear test. The intelligent inverse algorithm program, including a single-objective genetic algorithm program, a finite element parametric modeling program, and a UMAT subroutine were developed for numerical simulations. The simulation results were compared with the test data to verify the ability of the proposed modeling approach for analyzing the cyclic tensile behavior of the woven PVC-coated films in the tension–shear coupling state.

## 2. Cyclic Tensile Test

### 2.1. Samples

**Figure 1:**
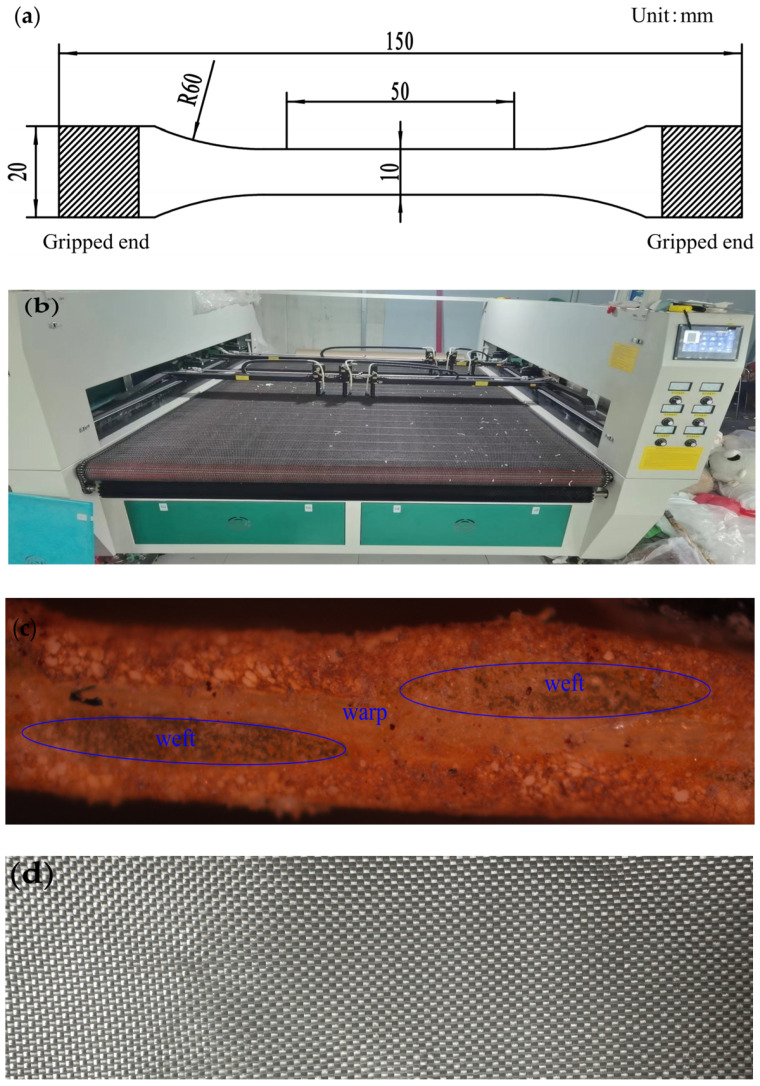
(**a**) Sample size diagram. (**b**) Laser cutting machine. (**c**) Weft direction cross-section. (**d**) Polyester fiber picture.

The tested material was woven PVC-coated film, provided by Shanghai Weibo Sunshade Facilities Co., Ltd. (Shanghai, China). The specific material parameters are shown in Table 1. The PVC-coated film fabric was made of polyester fiber yarns, and the yarn density was 1000 D. The test samples were processed according to the ISO 527-2 test standard [27], and the sample size is shown in Figure 1a. The gauge length of the specimen was 50 mm. The size file of a cyclic tensile specimen was established in the 2D software Autocad 2021 and saved in dxf format. Then, the dxf file was imported into the laser cutting machine to process the test sample. The laser cutting machine is supplied by Kunshan Jinheng Laser Technology Co., Ltd. (Kunshan, China). The model of the machine was JH-1823S2T6, the working voltage was 220 V, and the rated power was 2500 W, as shown in Figure 1b. The RX50M SOPTOP microscope, provided by Ningbo Shunyu Instrument Co., Ltd. (Ningbo, China), was used to observe the cross-section of the woven PVC-coated film, as shown in Figure 1c. Figure 1d is a picture of the polyester fiber.

**Figure 2:**
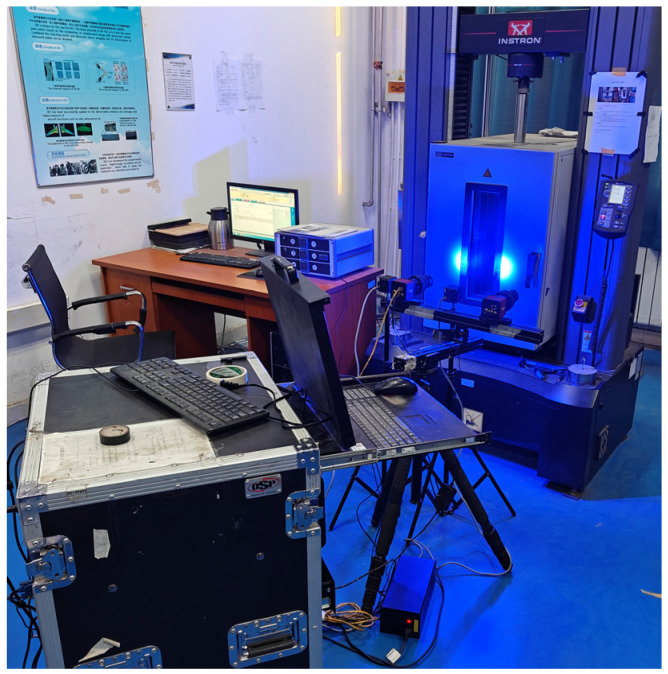
High-temperature DIC cycle tensile test.

**Figure 3:**
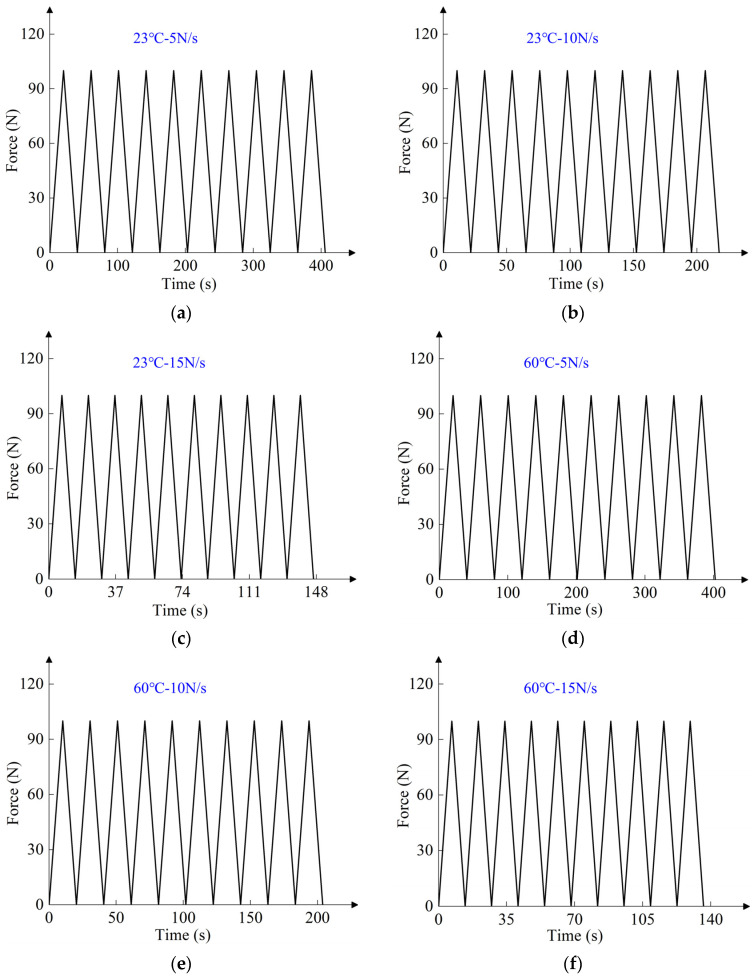
Cyclic loading spectrum at different temperatures and loading rates: (**a**–**c**) at 23 °C and different loading rates; (**d**–**f**) at 60 °C and different loading rates.

**Figure 4:**
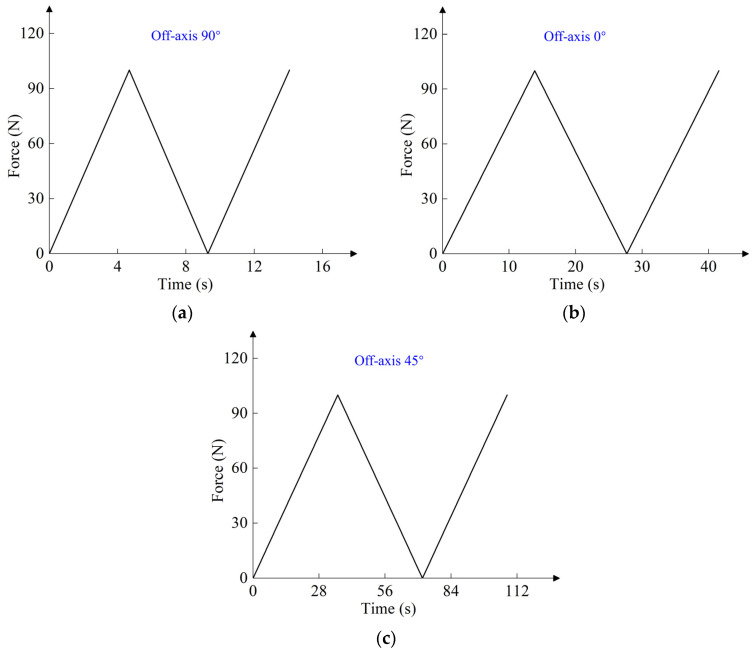
Cyclic loading spectrum of different off-axis angles at 23 °C and 20 mm/min loading rate: (**a**) off-axis 90°; (**b**) off-axis 0°; (**c**) off-axis 45°.

Cyclic tensile tests were carried out using the INSTRON 5982 testing machine. Figure 2 shows the high-temperature DIC cycle tensile test. The digital image VIC-2D 6.2.0 software was used to measure the variation of the gauge length during cyclic stretching at different temperatures. The nominal stress was obtained by dividing the load by the initial cross-sectional area of the gauge section. The true stress and true strain were obtained by converting *σ* = *σ*_b_(1 + *ε*_b_) and *ε* = ln(1 + *ε*_b_). *σ*_b_ and *ε*_b_ are the nominal stress and nominal strain, respectively. In Section 3, the cyclic tensile test was controlled by force loading rate, and the test specimens were the meridional specimens. The loading rates were 5 N/s, 10 N/s, and 15 N/s, and the temperatures were 23 °C and 60 °C. The cyclic loading spectrum at different temperatures and loading rates is shown in Figure 3. In Section 4, the cyclic tensile test was controlled by displacement loading rate. The loading rate was 20 mm/min, and the temperature was 23 °C. The test specimens were off-axis tensile specimens with off-axis angles of 0°, 45°, and 90°. The off-axis angle was relative to the weft direction. The cyclic loading spectrum at 23 °C and 20 mm/min loading rate is shown in Figure 4.

In both loading modes, the film was loaded to 100 N at different loading rates and then unloaded to 0 N at the same rate. There were two test pieces under each loading condition. As shown in Figure 3, each test piece was cyclically stretched ten times. As shown in Figure 4, each test piece was cyclically stretched one time. The obtained elastic modulus for each sample was compared with the mean elastic modulus, and only the sample data with modulus closest to the mean elastic modulus were selected for the picture presentation.

### 2.2. Experimental Results and Discussion

**Figure 5:**
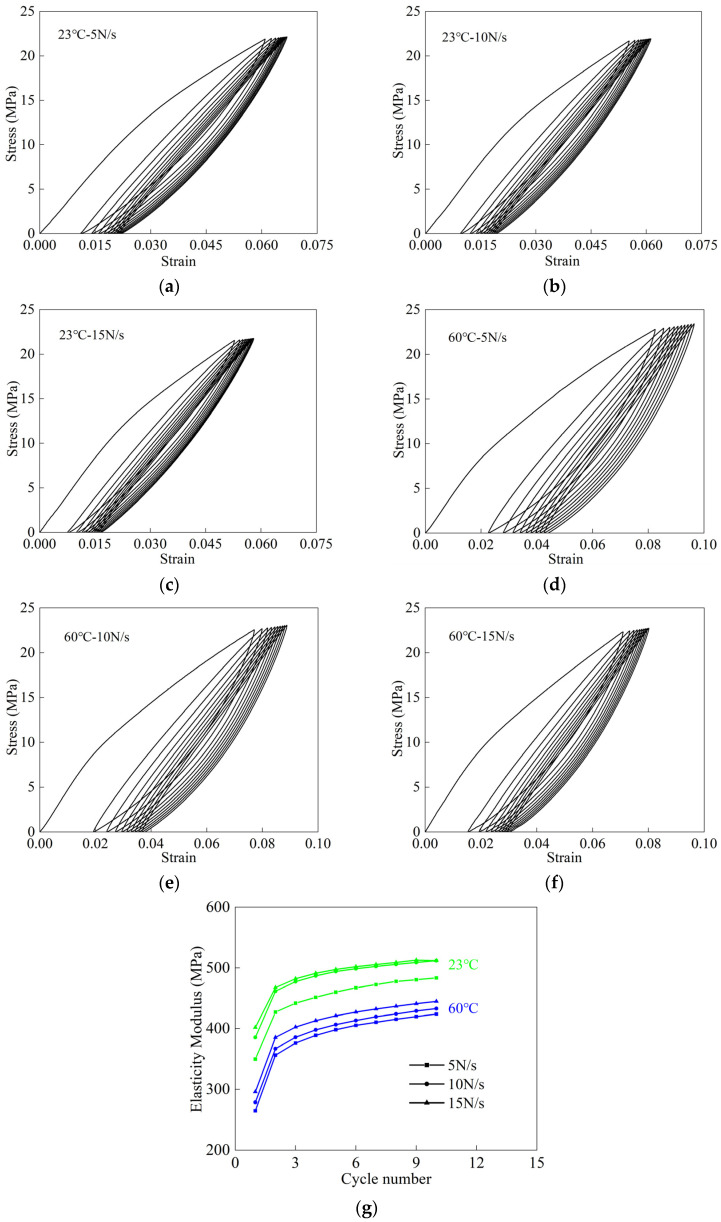
Cyclic tensile data under different loading conditions: (**a**–**c**) at 23 °C and different loading rates; (**d**–**f**) at 60 °C and different loading rates; (**g**) comparison of elastic modulus.

This section discusses the experimental data in Section 3. The true stress–strain curves for cyclic stretching under different loading conditions are shown in Figure 5. The initial stage of the curve was linear, the fiber cloth and the coating deformed together, and this stage appeared for a short time. Then, the curve entered a nonlinear stage, the coating cracked and separated from the fiber, and the warp yarn of the fiber cloth was gradually straightened. Therefore, the tensile stiffness decreased gradually in the nonlinear stage. After that, the loading curve entered the stage of quadratic linear elasticity, and the fiber cloth bore the load. This stage shows the elastic behavior of the fiber cloth.

The elastic modulus obtained by linear fitting is shown in Figure 5g. As the number of cycles increased, the elastic modulus increased gradually, but the increase rate decreased gradually. This indicates that the mechanical properties of the woven PVC-coated films tend to stabilize. This phenomenon is caused by the curl interchangeability of the woven structure. Curl interchange is a typical feature of fabric structure in the deformation process. As shown in Figure 5, this feature dominated in the first cycle. With the increase of the number of cycles, the curl interchange gradually disappeared. Comparisons shown in Figure 5g indicate that the elastic modulus of woven PVC-coated film was more sensitive to temperature. With the increase of temperature, the elastic modulus decreased gradually. At the same temperature, the elastic modulus increased with the increase of loading rate.

**Figure 6:**
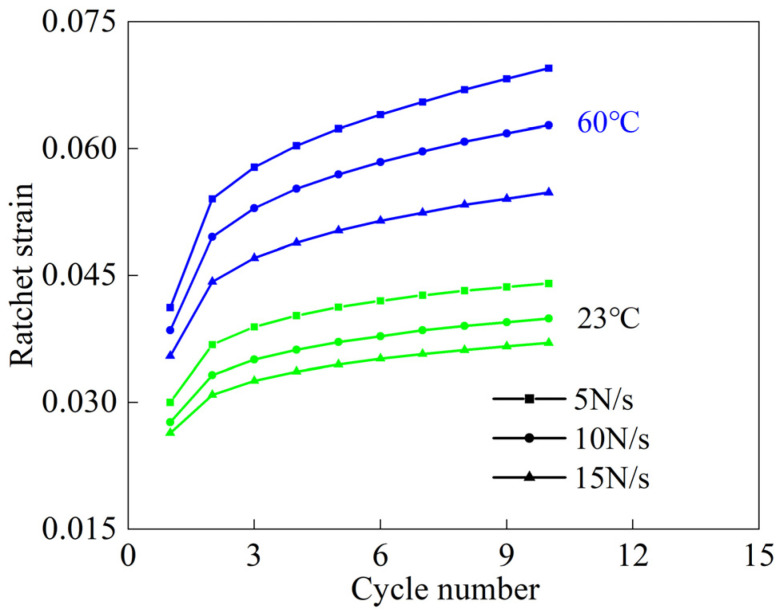
Ratchet strain under different loading conditions.

Plastic accumulation occurs in woven PVC-coated films under cyclic loading. The ratchet strain can be utilized to reflect the plastic cumulative deformation during the experiment. The ratchet strain refers to the mean value of maximum strain and minimum strain in a cycle [18,28]. As shown in Figure 6, the increase rate of ratchet strain decreased and eventually stabilized as the number of cycles increased. A similar phenomenon was reported in [28]. This was the same trend as that of the elastic modulus shown in Figure 5g. The main reason for this was the gradual accumulation of plastic deformation with the increase in the number of cycles, which eventually led to hardening. However, the plastic accumulation eventually led to the non-closure of the hysteresis loop. In addition, it can be noticed that the influence of temperature on ratchet strain was greater than that of loading rate. Moreover, the ratchet strain decreased with increasing loading rate. This was due to the fact that a lower loading rate provides enough time for the woven PVC-coated film to recover from deformation. Compared with the changing law of elastic modulus with the temperature and loading rate, the variation law of ratchet strain with the temperature and loading rate was opposite to that of the elastic modulus.

## 3. One-Dimensional Model of Woven Coating Films

### 3.1. Burgers Constitutive Model

When the woven PVC-coated film is stretched, obvious viscosity is seen, which produces unrecoverable plastic deformation. Therefore, in order to accurately describe the cyclic tensile properties of woven PVC-coated films, the influence of viscosity must be considered.

**Figure 7:**
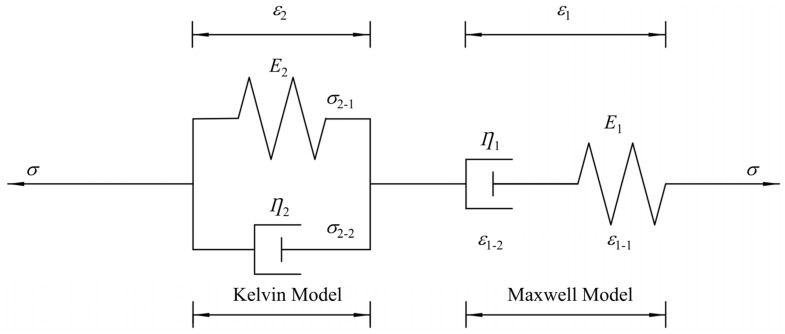
Structure diagram of the Burgers model.

For viscoelastic analysis of materials, a combination model of different types of the Maxwell model and Kelvin model, such as the Burgers model, is generally used. The Burgers model consists of a combination of the Maxwell model and the Kelvin model, as shown in Figure 7, and it is used to predict the deformation of woven PVC-coated films under different cyclic loading conditions. The differential constitutive equation of Burgers model is as follows:

(1) $$\sigma +p_{1}\sigma^{\prime }+p_{2}\sigma^{\prime \prime }=q_{1}\varepsilon^{\prime }+q_{2}\varepsilon^{\prime \prime }$$

where *p*_1_ = (*ƞ*_1_/*E*_1_) + (*ƞ*_1_ + *ƞ*_2_)/*E*_2_, *p*_2_ = (*ƞ*_1_*ƞ*_2_)/(*E*_1_*E*_2_), *q*_1_ = *ƞ*_1_, *q*_2_ = *ƞ*_1_*ƞ*_2/_*E*_2_. *σ*, *σ*′, and *σ*″ are stress, the first derivative of stress, and the second derivative of stress, respectively; *ε*, *ε*′, and *ε*″ are strain, the first derivative of strain, and the second derivative of strain, respectively; *E*_1_ and *E*_2_ are the elastic modulus; and *ƞ*_1_ and *ƞ*_2_ are the viscosity coefficients.

The initial conditions are *σ*(0^+^) = *E*_1_*ε*_1-1_(0^+^) = *E*_1_*ε*(0^+^), *ε*_2_(0^+^) = 0, and *ε*_1-2_(0^+^) = 0. Substituting the initial conditions into Equation (1) yields another form of the Burgers model as follows,

(2) $$\left\{\begin{array}{l} \sigma (0^{+})=E_{1}\varepsilon (0^{+})\\ \sigma^{\prime }(0^{+})=E_{1}\varepsilon^{\prime }(0^{+})-E_{1}^{2}(\frac{1}{\eta_{1}}+\frac{1}{\eta_{2}})\varepsilon (0^{+})\\ \sigma +p_{1}\sigma^{\prime }+p_{2}\sigma^{\prime \prime }=q_{1}\varepsilon^{\prime }+q_{2}\varepsilon^{\prime \prime } \end{array}\right.$$

In order to predict the stress–strain relationship of woven PVC-coated film during cyclic tensile loading, one cycle is divided into a loading stage and unloading stage. The loading phase and unloading phase can be represented by the conversion of Equation (2).

#### 3.1.1. Loading Phase

Since the Burgers model is composed of the Maxwell model and the Kelvin model, the strain of the Burgers model *ε*(*t*) can be obtained by superimposing *ε*_1_(*t*) of the Maxwell model and *ε*_2_(*t*) of the Kelvin model.

Substituting the loading stress *σ* = *h*(*t*) = *vt* (*v* is the loading rate) into the constitutive equation of the Maxwell model, integrating the resulting Maxwell model formula and applying the initial conditions (*t* = 0, *ε*_1_(0) = 0) yields

(3) $$\varepsilon_{1}(t)=\frac{v}{2\eta_{1}}t^{2}+\frac{v}{E_{1}}t$$

where *ε*_1_(*t*), *ƞ*_1_, and *E*_1_ are the strain, viscosity coefficient, and elastic modulus of the Maxwell model, respectively.

Substituting *σ* = *h*(*t*) = *vt* into the Kelvin model gives

(4) $$\varepsilon_{2}(t)=me^{-\frac{E_{2}}{\eta_{2}}t}+\frac{v}{E_{2}}t-\frac{v\eta_{2}}{E_{2}^{2}}$$

where *m* is the model parameter.

Applying the initial conditions (*t* = 0, *ε*_2_(0) = 0) into Equation (4) results in

(5) $$\varepsilon_{2}(t)=\frac{v\eta_{2}}{E_{2}^{2}}(e^{-\frac{E_{2}}{\eta_{2}}t}-1)+\frac{v}{E_{2}}t$$

A prediction model *ε*(*t*) of the loading phase can be obtained by combining Equation (3) and Equation (5). Since cyclic stretching is a linear loading process, the final form of model for *ε*(*t*) in the loading phase can be obtained by bringing *t* = *σ*/*v* into *ε*(*t*) as follows,

(6) $$\varepsilon (t)=\frac{\sigma^{2}}{2v\eta_{1}}+\frac{\sigma }{E_{1}}+\frac{v\eta_{2}}{E_{2}^{2}}(e^{-\frac{E_{2}}{v\eta_{2}}\sigma }-1)+\frac{\sigma }{E_{2}}$$

#### 3.1.2. Unloading Phase

The derivation process of the Burgers prediction model in unloading stage is similar to that in the loading stage. Combined with the initial conditions (*t* = *t*_0_, *ε*(*t*_0_) = *ε*_0_, *σ*(*t*_0_) = *σ*_0_), the final stress–strain relationship of the unloading model can be obtained:

(7) $$\varepsilon (t)-\varepsilon_{0}=-\frac{{\left(\sigma -\sigma_{0}\right)}^{2}}{2v\eta_{1}}+\frac{\sigma -\sigma_{0}}{E_{1}}-\frac{v\eta_{2}}{E_{2}^{2}}(e^{\frac{E_{2}}{v\eta_{2}}(\sigma -\sigma_{0})}+1)+\frac{\sigma -\sigma_{0}}{E_{2}}$$

**Figure 8:**
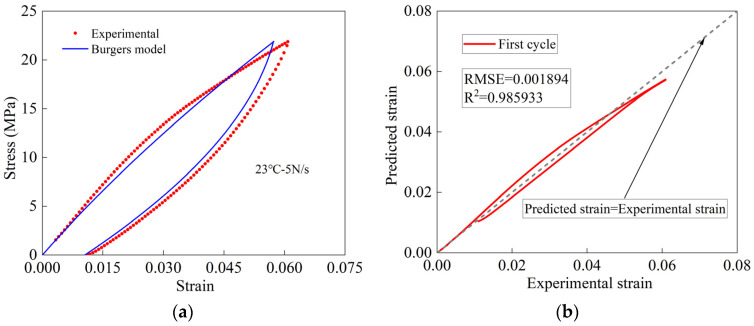
(**a**) A comparison of results obtained by the Burgers model and experiment. (**b**) Error analysis results.

The loading and unloading prediction models of the Burgers model are used to analyze the first cyclic stretching test data of woven PVC-coated film, and the results are shown in Figure 8. The prediction accuracy of the model is good in the initial loading stage, but the prediction becomes worse and worse in the later loading stage. The maximum stress value at the end of loading is less than the test result. In the unloading stage, the model can reflect the nonlinear deformation of the material well, but the coincidence degree between the predicted curve and the experimental curve is rather poor. The reason Is that there Is a deviation between the predicted Initial unloading point and the test initial unloading point.

The results show that the Burgers model has low accuracy in predicting the viscoelastic deformation of woven PVC-coated membrane. The Burgers model reflects only the ideal viscoelasticity, since an assumption is made that the elastic characteristics and viscosity characteristics of the material in the deformation process are equally proportional. The material studied in this paper, however, is a composite material with nonlinear viscosity and orthotropic behaviors. In the deformation process, the proportion of elastic and viscous characteristics of the material is not the same. Therefore, the improvement on the Burgers model needs to be made to accurately predict the mechanical behavior of woven PVC-coated film.

### 3.2. Improved Burgers Constitutive Model

The Burgers model holds that the viscosity coefficient *ƞ*_1_ is a constant. In fact, the viscoelastic deformation of woven PVC-coated film during the cycle includes non-recoverable deformation. Therefore, the viscosity coefficient *ƞ*_1_ in the Maxwell model should not be a constant and vary nonlinearly with time. It is expressed by $\eta_{1}=a_{1}e^{a_{2}t}$. The introduction of $a_{1}e^{a_{2}t}$ changes the proportion of *ƞ*_1_ and *ƞ*_2_ in the Burgers model. For *E*_1_ and *E*_2_, the ratio of *E*_2_ is adjusted using *a*_6_. Then, the Burgers model is corrected using *a*_3_, *a*_4_, and *a*_5_. The expressions of the new Burgers model for the loading and unloading phases are as follows:

(8) $$\varepsilon (t)=\frac{v}{a_{1}a_{2}^{2}}-\frac{v}{a_{1}}(-a_{3}\frac{\sigma }{a_{2}v}+\frac{1}{a_{2}^{2}})e^{-a_{2}(-a_{4})\frac{\sigma }{v}}+\frac{\sigma }{E_{1}}+\frac{v\eta_{2}}{{\left(a_{6}E_{2}\right)}^{2}}(e^{-\frac{a_{6}E_{2}}{v\eta_{2}}\sigma }-1)+a_{5}\frac{\sigma }{a_{6}E_{2}}$$

(9) $$\varepsilon (t)-\varepsilon_{0}=-\frac{v}{a_{1}a_{2}^{2}}+\frac{v}{a_{1}}(a_{3}\frac{\sigma -\sigma_{0}}{a_{2}v}+\frac{1}{a_{2}^{2}})e^{-a_{2}a_{4}\frac{\sigma -\sigma_{0}}{v}}+\frac{\sigma -\sigma_{0}}{E_{1}}-\frac{v\eta_{2}}{{\left(a_{6}E_{2}\right)}^{2}}(e^{\frac{a_{6}E_{2}}{v\eta_{2}}(\sigma -\sigma_{0})}-1)+a_{5}\frac{\sigma -\sigma_{0}}{a_{6}E_{2}}$$

where *a*_1_, *a*_2_, *a*_3_, *a*_4_, *a*_5_, and *a*_6_ are the model parameters.

**Figure 9:**
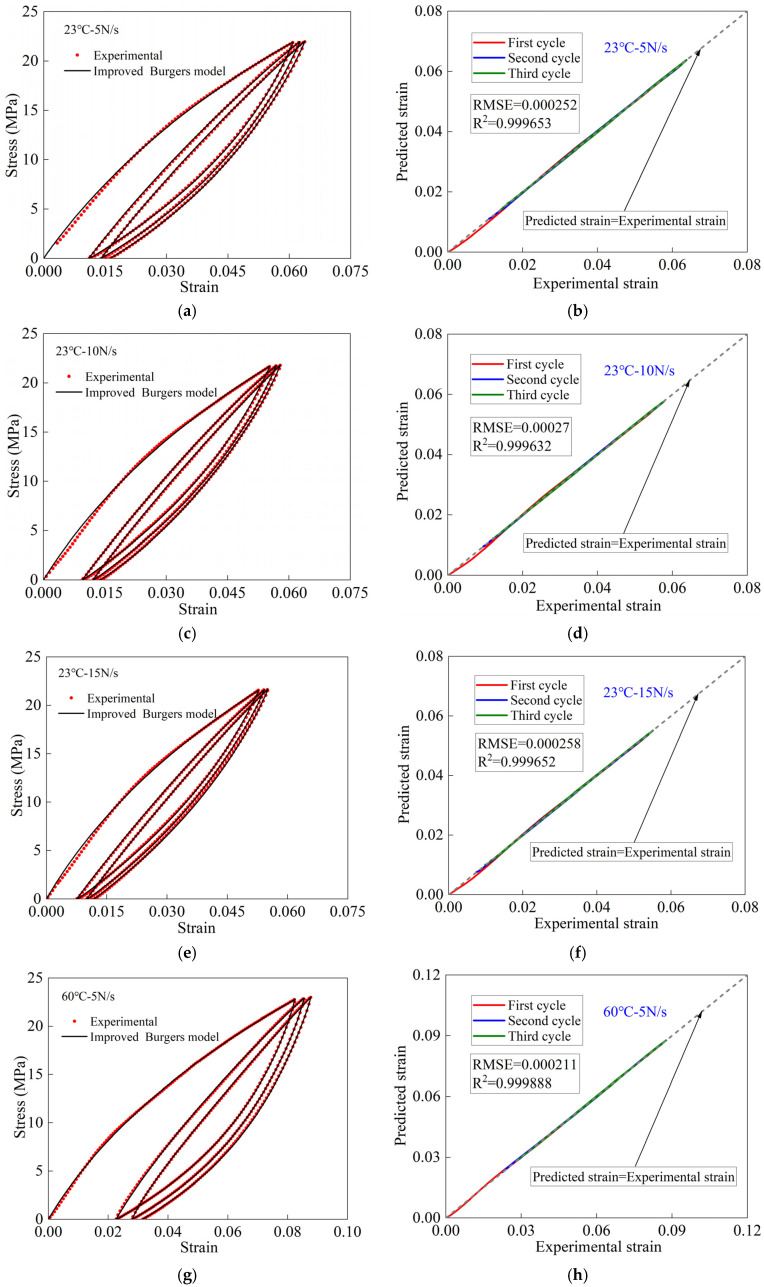

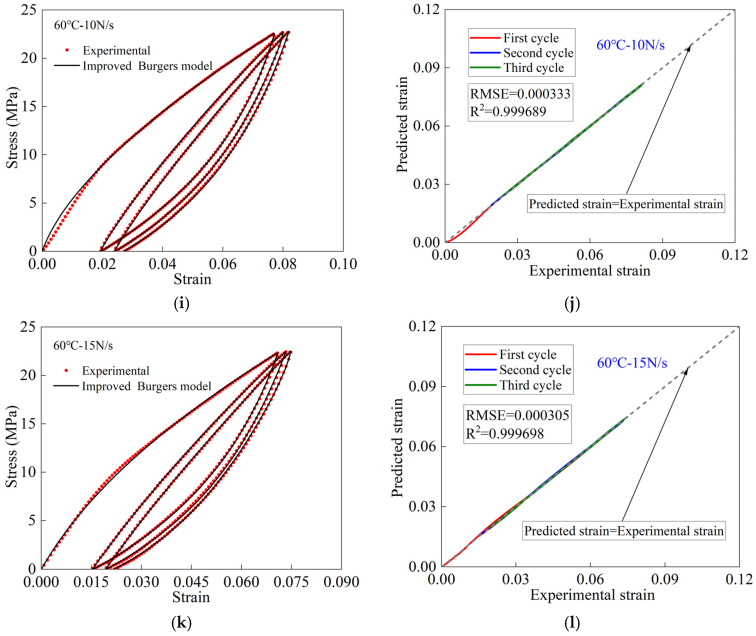
Comparisons of results between the improved Burgers model and test under different loading and temperature conditions: (**a**–**f**) at 23 °C and different loading rates; (**g**–**l**) at 60 °C and different loading rates.

The global search optimization algorithm is used to solve the model parameters of the improved Burgers model. Then, the improved Burgers model is utilized to predict the mechanical deformation of PVC-coated woven films during cyclic stretching. The first three cycle data of the cyclic tensile test were selected for comparison, as shown in Figure 9. In Figure 9e,i, the data predicted by the improved Burgers model for the initial loading phase of the first cycle showed small deviations from the experimental ones. However, during the remaining deformation, the predicted curves of the new model had a good coincidence with the experimental curves. The main reason for this deviation is experimental errors. As shown in Figure 9a,c,g,k, the new model was able to accurately depict the deformation process of woven PVC-coated films. Figure 9b,d,f,h,j,l shows the error analysis results. The comparative results in Figure 9 show that the improved Burgers model possesses good prediction accuracy for unloaded deformation under different loading conditions. This is because the improved Burgers model accurately captures the maximum and minimum stress points in each cycle. This is an important condition for the model to accurately predict the mechanical deformation during unloading.

## 4. Two-Dimensional Model of Woven Coating Films

### 4.1. Constitutive Model

As a one-dimensional model, the Burgers model cannot accurately describe the two-dimensional deformation of woven PVC-coated films. In this section, a macroscopic orthotropic nonlinear constitutive model is established. The relationship between stress and strain is described by polynomials. To characterize the elastoplastic deformation of woven PVC-coated film, the specific polynomial forms are introduced,

(10) $$\sigma_{w}(\varepsilon_{1})=A_{1}\varepsilon_{1}+A_{2}\varepsilon_{1}^{2}+A_{3}\varepsilon_{1}^{3}+A_{4}\varepsilon_{1}^{4}+A_{5}\varepsilon_{1}^{5}+A_{6}\varepsilon_{1}^{6}$$

(11) $$\sigma_{f}(\varepsilon_{2})=B_{1}\varepsilon_{2}+B_{2}\varepsilon_{2}^{2}+B_{3}\varepsilon_{2}^{3}+B_{4}\varepsilon_{2}^{4}+B_{5}\varepsilon_{2}^{5}+B_{6}\varepsilon_{2}^{6}$$

where *A*_1_–*A*_6_ and *B*_1_–*B*_6_ are the polynomial coefficients. The right hand of Equations (10) and (11) is called warp and weft (fill) polynomials.

The tangent modulus in warp and weft directions is obtained by differentiating the two polynomials separately. The tangent modulus in direction “1” and direction “2” is expressed as

(12) $$E=E_{w}(\varepsilon_{1})=A_{1}+2A_{2}\varepsilon_{1}+3A_{3}\varepsilon_{1}^{2}+4A_{4}\varepsilon_{1}^{3}+5A_{5}\varepsilon_{1}^{4}+6A_{6}\varepsilon_{1}^{5}$$

(13) $$E_{2}=E_{f}(\varepsilon_{2})=B_{1}+2B_{2}\varepsilon_{2}+3B_{3}\varepsilon_{2}^{2}+4B_{4}\varepsilon_{2}^{3}+5B_{5}\varepsilon_{2}^{4}+6B_{6}\varepsilon_{2}^{5}$$

while the shear modulus is represented by

(14) $$G_{12}=G_{wf}(\gamma_{12})=C_{1}+2C_{2}\gamma_{12}+3C_{3}\gamma_{12}^{2}$$

where *C*_1_–*C*_3_ are the polynomial coefficients. Equation (14) is called the shear polynomial for simplicity.

Substituting the three polynomials of Equations (12)–(14) into the two-dimensional orthotropic stiffness matrix obtains the new orthotropic nonlinear model of PVC-coated film.

### 4.2. Coefficient Acquisition

The model parameters in Equations (10) and (11) are determined by curve fitting the true stress–strain data of the warp and weft specimens, which are obtained by tensile tests at room temperature. However, the coefficients in Equation (14) are solved by the anti-inference method to circumvent performing the difficult shear test, although they can also be obtained by curve fitting the shear test data. The dimensions of the woven PVC-coated film specimens used for uniaxial tensile tests are the same as the one shown in Figure 1a. Table 2 shows the obtained parameters of the warp and weft polynomials. Then, based on Equations (12)–(14), a UMAT subroutine is written for numerical simulations.

The inverse algorithm is realized by a self-developed program together with ABAQUS 2021 software. The inverse algorithm program includes a single-objective genetic algorithm program, a finite element parametric modeling program, and a UMAT subroutine. The single-objective genetic algorithm program and the finite element parametric modeling program are written in Python, and the UMAT subroutine is written in FORTRAN.

Genetic algorithm is a bionic global search algorithm with strong random search ability. It mimics biological selection and evolution to obtain the best individuals. Selection, crossover, and mutation are three operators of genetic algorithm. The optimization steps of genetic algorithm are mainly divided into encoding, decoding, selection, crossover, and mutation [29].

(1) Chromosome encoding

In genetic algorithms, optimization variables are usually encoded onto the chromosomes in either real-coded or binary form. Genetic algorithms in binary form have a wider range of applications than in real-coded form. Therefore, in this paper, the binary form is used to encode the chromosomes.

**Figure 10:**
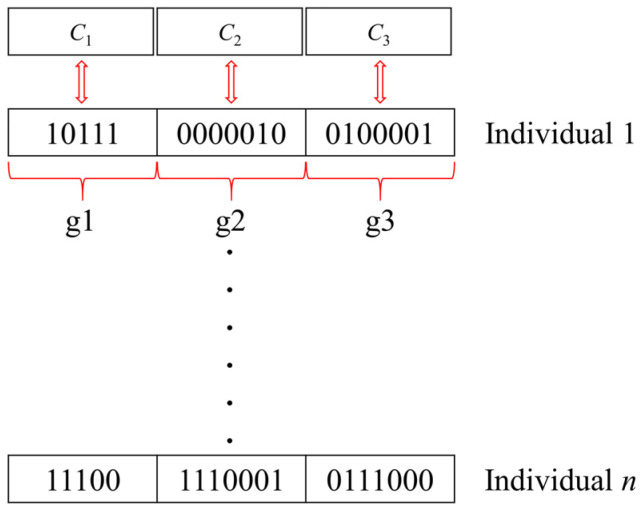
The encoding process of chromosomes.

As shown in Figure 10, during uniaxial stretching, each chromosome consists of three genes; *gi* (*I* = 1, 2, 3) denotes the length of each gene; and *gi* (*I* = 1, 2, 3) is used to denote the optimization parameters *C*_1_, *C*_2_, and *C*_3_, respectively.

(2) Fitness function

In the uniaxial stretching process of this paper, the objective function in the genetic algorithm program is the root mean square error (*RMSE*) of the simulated and experimental uniaxial tensile load-displacement data of woven PVC-coated films with a 45° off-axis. Its specific form is described,

(15) $$RMSE=\sqrt{\frac{\sum_{i=1}^{n}{\left(f_{i}^{s}-f_{i}^{e}\right)}^{2}}{n}}$$

where $f_{i}^{s}$ and $f_{i}^{e}$ are the *i*th simulated load value and test load value, respectively. *N* is the total number of load-displacement data points in the test.

In this paper, the fitness values of uniaxial stretching and uniaxial cyclic stretching were calculated from the fitness function of the form shown in Equation (16), which is mainly used to assess the competitiveness of individuals in the optimization process. The better the individuals in the population are, the higher the fitness value. The selection operator in the optimization algorithm is guided by the fitness value, so the evolutionary direction of the population is determined by the fitness function.

(16) $$F=\frac{1}{RMSE}$$

(3) Chromosome decoding

**Figure 11:**
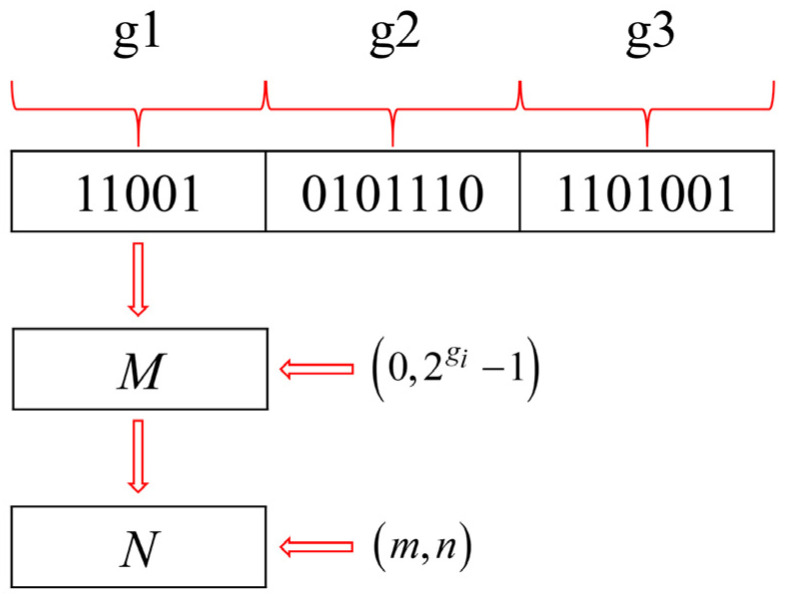
The decoding process of chromosomes.

The decoding process is to convert binary chromosomes into optimized variables, as shown in Figure 11. The binary gene at site *I* on a chromosome is first converted to a decimal number (*M*), and then the decimal number is converted to the actual optimization variable (*N*) in the search region (*m*, *n*) by

(17) $$N=m+(\frac{n-m}{2^{g_{i}}-1})\times M$$

In the optimization process, the optimization efficiency is affected by the search step size (*S*). The definition of search step size *S* is given below:

(18) $$S=\frac{n-m}{2^{g_{i}}-1}$$

It can be found that the search step size is affected by *m*, *n*, and *gi*. A reasonable search step size will improve the optimization efficiency and obtain the desired optimization parameters. Therefore, the choice of parameters (*m*, *n*, and *gi*) for the search step size needs to be careful.

(4) Chromosome selection

During chromosome selection, by comparing fitness values, excellent individuals are retained, and poor individuals are eliminated. In this way, good gene transmission is passed on to the next generation, ensuring the quality of the chromosome population in the offspring.

(5) Chromosome crossover

**Figure 12:**
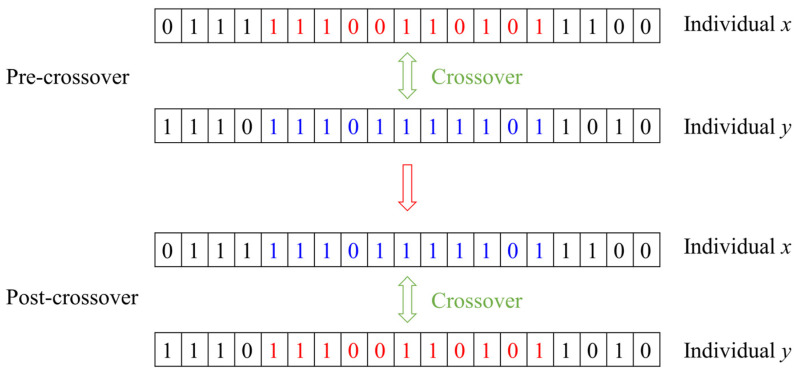
The crossover process of a chromosome.

According to the crossover probability, the process of interchanging binary gene segments on the chromosomes of two parents in a population to produce two new chromosomes is known as chromosome crossover, as shown in Figure 12. During chromosome crossover, the location of the crossover point is chosen randomly. The generation of new chromosomes by crossover interchanges expands the diversity of the chromosome population and also increases the possibility of obtaining optimal parameters.

(6) Chromosome mutation

**Figure 13:**
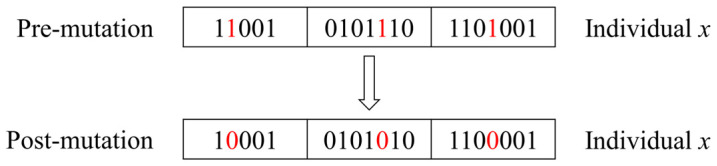
The mutation process of a chromosome.

Chromosome mutation is the conversion of the binary number 0 to 1 or 1 to 0 of a gene segment on a chromosome, depending on the probability of the mutation, resulting in a new chromosome, as shown in Figure 13. The purpose of mutation, like crossover, is to expand the diversity of the chromosome population.

Using the newly developed inverse algorithm program, the initial values of the model parameters of the shear polynomial is not required, but only the range of three model parameters is needed. The two constraints in the optimization process are that the value of the shear polynomial is always positive and the validity of the simulation results. And the crossover and mutation probabilities are 90% and 10%, respectively.

**Figure 14:**
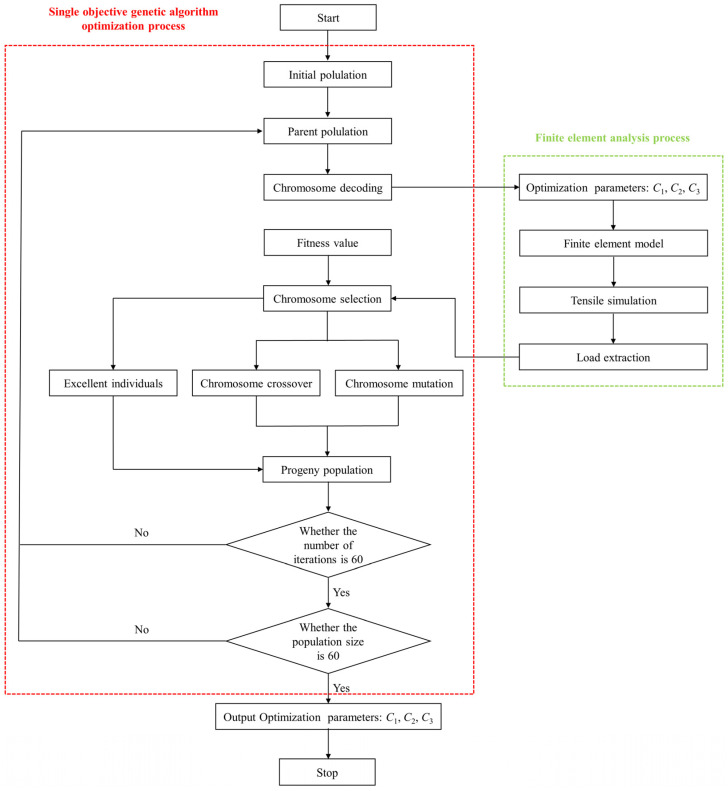
Inverse algorithm optimization process diagram.

Figure 14 shows the flow charts of the inverse algorithm optimization process for obtaining the three optimization parameters (*C*_1_, *C*_2_, and *C*_3_) in Equation (14). Both the number of population iterations and population size in the genetic algorithm are 60. The inverse algorithm developed in this paper selects the individual with a large fitness value by comparison. If the simulation results do not converge, the fitness function value of the computational instance is 0.00001, the computational instance is ignored, and the computation continues. Therefore, this inverse algorithm is intelligent and convenient. The obtained shear polynomial model parameters are listed in Table 3.

**Figure 15:**
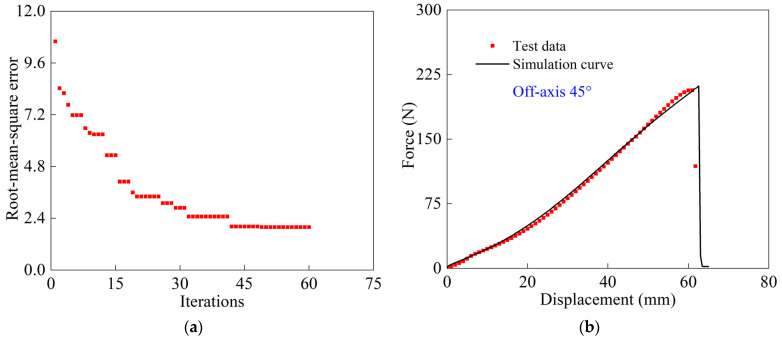
Results of the inverse algorithm for uniaxial stretching: (**a**) the root mean square error; (**b**) the comparison of test and simulated tensile load-displacement data of woven PVC-coated film with 45° off-axis.

In the single-objective genetic algorithm, the best individuals in the current population are passed to the next generation population through population iteration, and then the operation is repeated until the set population number is reached, and following this the analysis stops. Figure 15a shows the root mean square error under different iterations. It is seen that the root mean square error gradually decreases as the number of the population increases, and the final error is 1.99. Figure 15b shows a comparison of test and simulated load-displacement data for a woven PVC-coated film with an off-axis of 45° at room temperature. The agreement validates the obtained shear model parameters by the inverse algorithm.

### 4.3. Constitutive Model Validation

**Figure 16:**
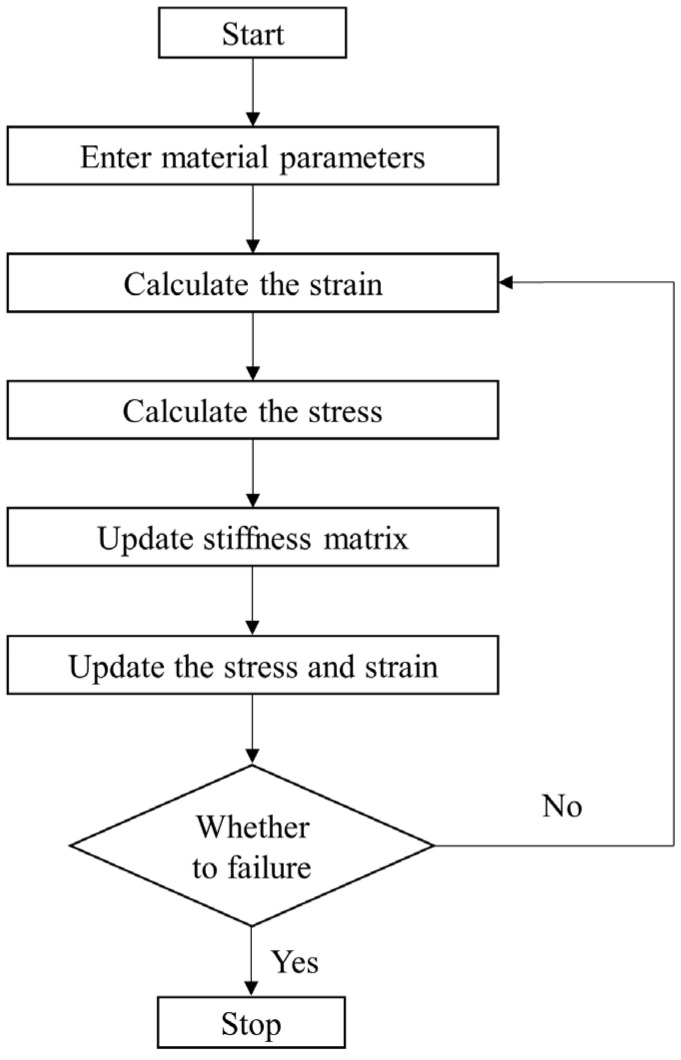
Flow chart of the uniaxial stretching UMAT subroutine.

The obtained model parameters are introduced into the UMAT subroutine to simulate the uniaxial stretching at different off-axis angles and room temperature using ABAQUS. The flow chart of the UMAT subroutine for uniaxial stretching is shown in Figure 16. The failure criterion of Tsai–Hill [19] is selected as the damage criterion.

(19) $$\frac{\sigma_{1}^{2}}{X^{2}}-\frac{\sigma_{1}\sigma_{2}}{X^{2}}+\frac{\sigma_{2}^{2}}{Y^{2}}+\frac{\tau_{12}^{2}}{S^{2}}=1$$

where *σ*_1_, *σ*_2_, and *τ*_12_ are the stress in the warp direction, the stress in the weft direction, and the shear stress, respectively.

**Figure 17:**
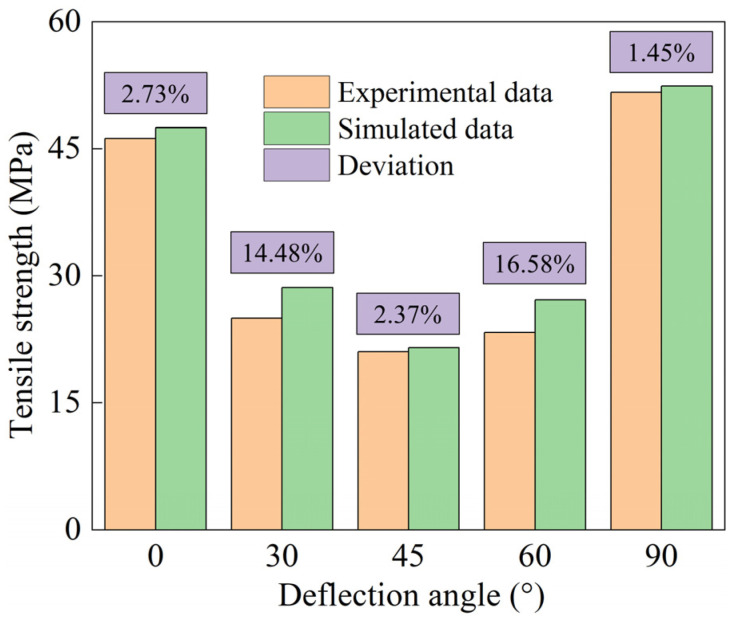
A comparison of tensile strength between the test and simulation.

During the progressive damage analysis, the components of the stiffness matrix are multiplied by 0.001 when the stress state meets the Tsai–Hill criterion. The comparison between simulated and experimental tensile strength is shown in Figure 17.

**Figure 18:**
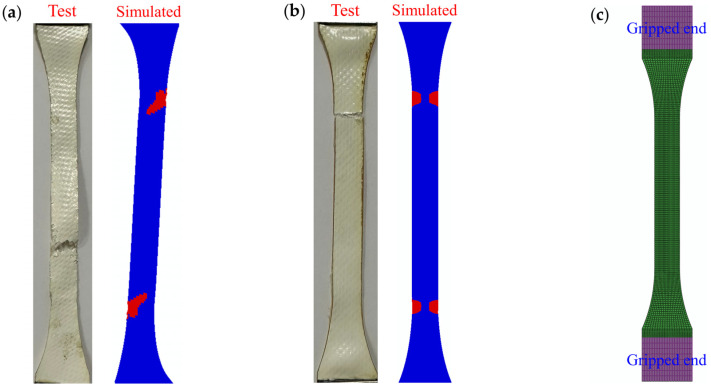
Comparison of experimental and simulated damage modes under different off-axis angles during uniaxial stretching: (**a**) 30°; (**b**) 90°; (**c**) finite element model.

As can be seen from Figure 17, the error between the simulated and experimental results was small at off-axis angles of 0°, 45°, and 90°. The simulation errors of 30° and 60° off-axis angles were large, and the maximum error was 16.58%. This phenomenon is mainly caused by experimental errors. Because the specimens with off-axis angles of 0°, 45°, and 90° are stressed more evenly during the tensile process, they are not sensitive to experimental errors. However, the specimens with off-axis angles of 30° and 60° will produce large shear stress during the test, which will accelerate the failure of the specimens. Therefore, a small off-axis error during specimen mounting will have a great impact on the experimental results. Although the simulation errors of 30° and 60° off-axis angles are relatively large, they are acceptable. Figure 18 shows that the distribution of failure elements is basically consistent with the failure mode of the off-axis test specimen. This further validates the correctness of simulations.

### 4.4. Uniaxial Cyclic Test Simulation

**Figure 19:**
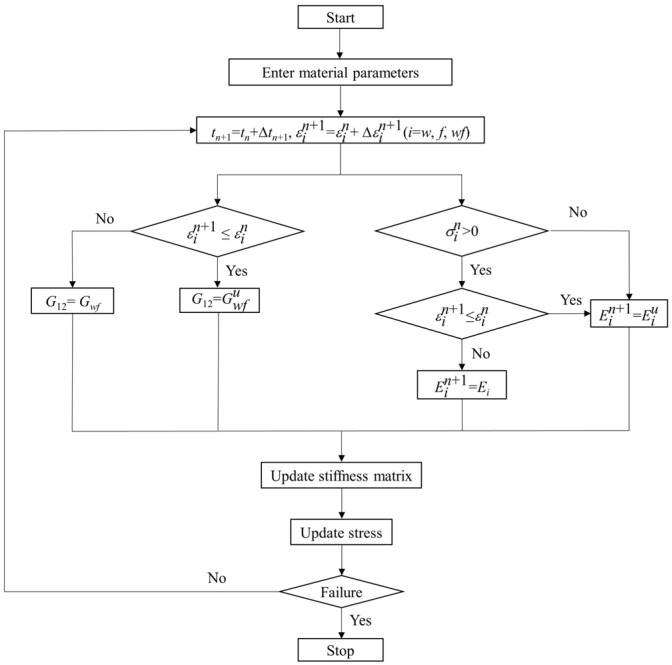
Flow chart of the cyclic stretching UMAT subroutine.

The warp and weft (fill) polynomial coefficients and shear polynomial coefficients of the woven PVC-coated film in the loading stage of the cyclic stretching test were the same as those of uniaxial stretching, as shown in Table 2 and Table 3. Based on the obtained model parameters in Section 4.2, a UMAT subroutine for uniaxial cyclic stretching simulation was developed. Figure 19 shows the flow chart of the loop stretching UMAT subroutine in detail. In Figure 19, $E_{i}^{u}$ (*I* = *w*, *f*) is the warp and weft unloading modulus, and $G_{wf}^{u}$ is the shear modulus during unloading. 

To simplify the simulation, the unloading modulus is assumed to be constant during unloading. Based on the true stress–strain data of cyclic tension, the unloading modulus values in the warp and weft directions are approximately the slopes of the curve between the initial unloading point and the initial reloading point. Based on the coefficients in Table 2 and Table 3 as well as the warp and weft (fill) unloaded modulus of woven PVC-coated film, the unloading shear modulus ($G_{wf}^{u}$) is determined by using the inverse algorithm to fit the cyclic test data of the off-axis 45° specimen. The program composition in the inverse algorithm is the same as the one in Section 4.2. However, the UMAT subroutine for uniaxial stretching needs to be replaced with one for cyclic stretching. The optimization steps are shown in Figure 14. The objective function in the genetic algorithm program is the root mean square error of the simulated and experimental cyclic tensile load-displacement data of woven PVC-coated films with a 45° off-axis. Table 4 summarizes the material parameters required to simulate the unloading process. As shown in Figure 4, the PVC-coated film is loaded to 100 N at a displacement loading rate of 20 mm/min during the test, and then unloaded to 0 N at the same displacement loading rate.

**Figure 20:**
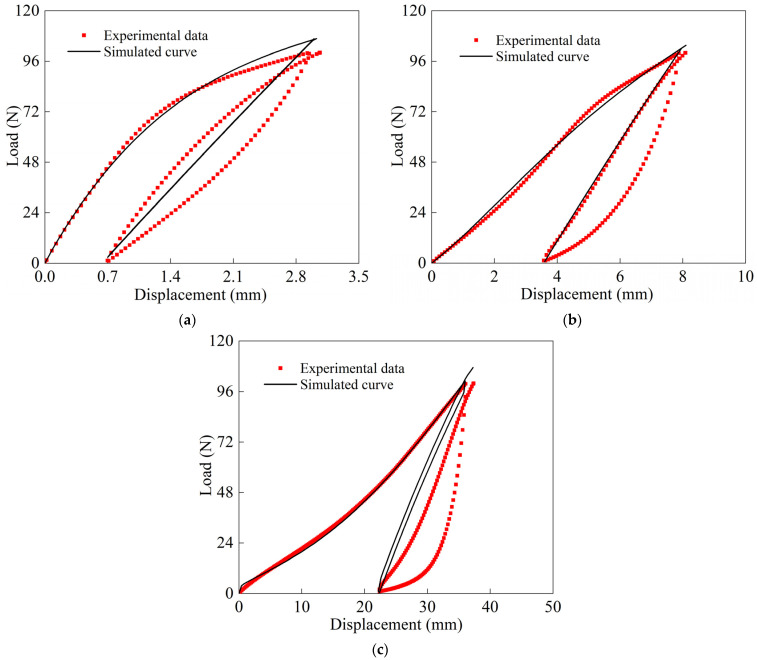
Comparisons of test and simulated cyclic tensile data of three off-axis angles at room temperature: (**a**) 90° (warp); (**b**) 0° (weft); (**c**) 45°.

A four-node reducing integral film element (M3D4R) was used to establish the finite element model of the specimen, and the simulation results are shown in Figure 20. In Figure 20a,b, compared with the test data, it is seen that the proposed orthotropic nonlinear model had good prediction ability for the cyclic tension of the warp and weft. It accurately reflected the tensile nonlinear behavior of the material’s spindle direction and the influence of loading history. The 45° off-axis specimen was in the tension-shear coupling state during the cyclic tensile process and thus had a complex deformation behavior. This is similar to the stress state of membrane structure in practical engineering. Figure 20c shows the forecast data of the 45° off-axis specimen. It is seen that the simulation results agreed well with the test results. This shows that the established 2D model can effectively analyze the cyclic tensile deformation under the tension–shear coupling state. It is seen that the simulation results for 45°off-axis cyclic tension were better than the ones for 0° and 90° off-axis cyclic tension. The reason is that the shear polynomial of the loading stage and the shear modulus of the unloading stage during the cycle loading were calibrated by the 45° off-axis test data. Moreover, the maximum simulation error was less than 7.2%, within the acceptable range. At the same time, the high precision of 45° off-axis also shows that the inverse algorithm can improve the accuracy of simulation results.

## 5. Conclusions

In this paper, the cyclic tensile deformation of woven PVC-coated film was studied by using the proposed 1D model and 2D model. The 1D model, an improved Burgers model, was established. The effects of the time-dependence of the viscosity coefficient and the ratio of elastic to viscous deformation were considered simultaneously. It can better predict the cyclic drawing process of woven PVC-coated fabrics at different temperatures than the Burgers model and accurately depict the stretching and unloading deformation during the cycle. The 2D model is an orthotropic nonlinear model, whose relationship between modulus and strain is characterized by polynomials.

UMAT subroutines of off-axis stretching and off-axis cyclic stretching are written separately. The warp and weft polynomial coefficients of the 2D model are obtained by curve fitting the tensile test data, while the inverse algorithm is used to obtain the shear polynomial coefficients to circumvent performing the difficult shear test. The inverse algorithm program includes a parametric modeling program for a tensile specimen, a single-objective genetic algorithm program, and a UMAT subroutine. The cyclic stretching behavior with different off-axis angles is simulated and compared with the experimental result. Comparisons show that the proposed 2D orthotropic nonlinear model can accurately capture the cyclic tensile deformation of woven PVC-coated film.

At the same time, for the 45° off-axis, the 2D model figuratively simulates the hysteresis loop behavior, and the deformation trend of the hysteresis loop is similar to that of the test. Therefore, the newly developed 2D orthogonal nonlinear modeling method and inverse algorithm are effective. It can be used to analyze the cyclic tensile deformation of woven PVC-coated film material under the state of tensile–shear coupling with good prediction.

## Figures and Tables

**Table 1:** Material parameters of woven PVC-coated film.

Weight (g/m^2^)	Thickness (mm)	Tensile Strength (N/5 cm)		Weave Density (yarns/cm)	
		Warp	Weft	Warp	Weft
750	0.73	2400	2100	8.5	8.5

**Table 2:** Warp and weft polynomial coefficients of woven PVC-coated film at room temperature.

Warp		Weft	
Variable	Value (Mpa)	Variable	Value (Mpa)
*A*_1_	1654.15	*B*_1_	245.22
*A*_2_	−53,272.88	*B*_2_	4172.43
*A*_3_	975,249.21	*B*_3_	−89,829.62
*A*_4_	−9,123,857.73	*B*_4_	746,159.5
*A*_5_	42,965,800.57	*B*_5_	−2,649,681.06
*A*_6_	−79,551,644.73	*B*_6_	3,517,207.21

**Table 3:** Shear polynomial model parameters of woven PVC-coated film at room temperature.

Variable	Value (Mpa)
*C*_1_	9.11
*C*_2_	1.03
*C*_3_	55.28

**Table 4:** Material parameters of woven PVC-coated film during unloading at room temperature.

Variable	Value (Mpa)
$E_{w}^{u}$	998.72
$E_{f}^{u}$	575.95
$G_{wf}^{u}$	69.87

## Data Availability

Data are contained within the article.
